# Pyorrhea Alveolaris—With Some Notes from the Practice of the Author

**Published:** 1898-11

**Authors:** A. W. Harlan

**Affiliations:** Chicago, Ill.


ARTICLE IV.

### PYORRHEA ALVEOLARIS.—WITH SOME NOTES FROM THE PRACTICE OF THE AUTHOR.

A. W. HARLAN, A. M., M. D., D. D. S., CHICAGO, ILL.

Read before the New York Odontological Society, March 15, 1898.

It is no part of my plan to-night to read extracts from the papers of other writers for your benefit, as you have the same means of access to them that I have, and if you have not availed yourselves of the privilege it is no fault of mine.

We have been told that the ancients suffered from loosening of the teeth, and from recession of the gums and the wasting of alveoli, in fact, I have read somewhere that evidences of these facts have been seen by living human eyes when looking at the skulls of mummies and other cheerful specimens of bygone generations of men and women. I have seen these subjects in Washington, Cambridge, and elsewhere, so that I know that the ancients must have suffered in this way when mercury was not used as a medicine and salt pork was not an article of daily diet. Indeed, I believe that port wine or beer was not the ordinary daily beverage of some of the savages of bygone times.

So far as I know, women did not use sewing-machines, huddled close together in ill-ventilated rooms, nor did the men of those days chew tobacco or smoke pipes, or do book-keeping with electric lights to take the place of sunshine. There were no sleeping carriages, nor steam heat, nor razor-pointed shoes; then those people lived before there were dyspepsia pills or patent cures for gout or rheumatism. They did not have the blessed privilege of choosing from dozens of proprietary medicines, warranted to destroy various pus-producing cocci, whether they were prefixed with "staph" of "strep," or some other new fangled way of distinguishing them from others that are not pathogenic.

There were no forceps or separators, or floss silks, or files, or dental engines, or tooth brushes. Malnutrition and chewing gum had not been heard of, and the uric acid diathesis was not even dreamed of. People of those ancient times suffered from loose teeth and socketless teeth just the same, and still they were deprived of the valued service of stomatologists, dentists, and oral surgeons. The delicate needles of gold, platinum and steel, with the aseptic plungers, were not invented then, nor did our forefather dentists need them, as the slender push-and-pull scalers had not been evolved. There were no local and few general anesthetics, so that all they had to rely upon were astringents and detergents for laving their mouths.

Men and women lived close to nature in those remote ages, but, in spite of these facts, teeth became loose, and have continued to loosen until the present time. If we could only summarize all of the causes of loosening of teeth it is doubtful, even now, if we could in one or two short generations totally prevent such a disaster to the human race.

I have no new theory to present to you to-night, because I am not sure that the same causes are universal throughout the United States. In the mountains, where dampness does not exist and microbes do not thrive, the varieties of looseness of the teeth are not the same that we see on the borders of a great lake or in the lowland, where miasms flourish and people suffer from intermittent fevers and rheumatism.

In cosmopolitan cities, like New York, Philadelphia and Chicago, where the radical characters are so numerous, we must seek for different causes, largely influenced by hereditary tendencies. The robust, out-door workers, both men and women, do not seem to be so often attacked by loosening of the teeth as those whose occupations confine them to indoor life. Many of the prevalent diseases, like typhoid, the various forms of catarrhal and other inflammations of the mucous membrane, perhaps do, in many instances, furnish suitable fields for the ravages of the particular coccus which invades the territory surrounding the neck of the tooth.

The administration of medicines, frequently not suited to a case, may be responsible for much of the irritation and consequent detachment of the pericementum from the neck of the tooth. It is certain that many cases of loosening are due to a crowded condition of the teeth, and it is equally certain that many others are due to the unwise extraction of teeth and the fitting of partial dentures to the mouth not suitable as substitutes for the teeth already lost. Badly made contour fillings, and those improperly finished, are a frequent cause of the detachment of the gingival margin of the gums; neglect, uncleanliness, the formation of salivary deposits, pernicious habits of eating and drinking, lack of exercise, the abuse of toothpicks and thread, disks, files, clamps, and poorly fitting separators and regulating appliances, all have something to do with the inception of the initial lesion.

I am not one of those who believe that gout and rheumatism can be regarded as potent factors in causing loosening of the teeth. All those teeth are not incrusted with deposits on their roots. Many, it is true, have deposits on them, but not all. In framing a system of treatment each case must be studied for the cause, and, if it be found, the prognosis may be favorable or not, as the history of the case will disclose.

Too little attention is paid to the care of the teeth of families by the dental surgeon himself. Incipient pyorrhea is not checked soon enough, and sooner or later the practitioner is brought face to face with a condition of things not desired or easily controlled by him. We find that our regular systematic clientele are not the frequent sufferers, but those migratory people who are never satisfied with the conscientious care of a family dentist, but who flit from office to office for reasons of their own, until gradually their teeth are all, or nearly all, in a hopeless state of chronic looseness. (Some do not consult a dentist at all until their teeth are hopelessly loose.)

I do not think it proper to consider syphilis as an etiological factor in producing looseness of the teeth, although perhaps a small percentage of the cases seen may be traced to such a cause or to the heroic medication of the patient during the active stages of this disease.

It seems to me that Witzel was nearer right in calling loosening of the teeth "infectious alveolitis" than any of our modern authors.

In presenting these thoughts to you I am not unmindful of the strong claims to a name presented by Magitot, "symptomatic alveolar arthritis;" Black, "phagedenic pericementitis," and the late Dr. Riggs, "Riggs' disease;" "Ingersoll, "alveolar ulceration," and "Wedl, "pyorrhea alveolaris." A few names have been presented by other authors, but for our present purpose we incline to the latter as more fully expressing what is meant by an author when he talks of ulceration of the socket and coincident loosening of teeth. "What's in a name?" Is it not true that many of the causes of loosening of teeth already mentioned are grouped together in one mouth, and the patient may at the same time be suffering from some intercurrent malady not responsible for the appearance of the gums? Some of the lesions of the gingivæ are so simple, if seen in the beginning, that little or no treatment will cause them to disappear; and still if one or more teeth are missing we find some of them migrating for support against a more firmly fixed tooth; aided frequently by abnormal mouth-breathing; which is a potent factor in loosening, yet little regarded in many cases. Any cause, obesity, occulated or malformed nasal passages, or growths in the past-nasal region, will be sufficient to induce mouth-breathing. In such cases the teeth spread and project, and pockets are formed around the roots; usually on the lingual aspects at first, but later they are seen encircling the whole root.

When my attention was first drawn to the loosening of teeth, in a constantly growing practice, about 1878, I began a series of observations as to the causes most prominently concerned in bringing about such phenomena. I was very soon impressed with the idea that the mere deposit had little to do with what we deem pyorrhea alveolaris. It is true that many teeth are loosened and lost from accumulations of tartar or calculi on the tooth, roots, and crowns, but we do not find deep pouchets or pockets alongside the roots when such deposits alone are found on the teeth. The removal of such deposits, the use of astringent and stimulating washes, will soon re-establish a good color and firmness to the gums. That heredity played an important part in the causation of looseness of the teeth became firmly fixed in my mind; that we could not ascribe all loosening of teeth to one or two causes, the abuse of liquors, a salt meat diet, mercury, and not even to gout or rheumatism, or both, could we ascribe even a small percentage of these cares, this is true at least in Chicago, where all of my labors have been confined. The mere fact of a tooth being deprived of the pulp is not cause sufficient for the tooth to become loose; indeed, its destruction in many cases is imperatively demanded to permit the tooth remaining in the mouth. As I was unable to group causes to cover all cases. I concluded to study them individually, with the following results :

First mercurical stomatitis. In all of these cases there is a symmetrical detachment of the peridental membrane, which yields to a treatment of cleansing the roots and the injection of trichloracetic acid every second day around the individual tooth, and the frequent use of a boro-glycerol solution. ten per cent., in water.

Second, syphilis. Thorough removal of the deposits and a potassium chlorate solution, five per cent. in water, used frequently, five or ten times daily, and the administration of ten-grain doses of potassium iodid three times a day, after meals, in peppermint water, is generally all that is required. In all other cases the general rule is to remove deposits and necrosed bone thoroughly, and then inject the pouches or pockets with, first, for one week, a bichlorid solution, one to one thousand, made with hydrogen peroxid, say one grain to two ounces of the peroxid, and five grains of tartaric acid. Later I use at first a strong solution of trichloracetic acid for two or three visits, about five to eight per cent. in distilled water. When I find that the case is doing well, say at the end of two or three weeks, I begin to use about a five per cent. solutiou of alumnol, in water, to which is added about three per cent. of resorcin.

I usually flavor this with oil of wintergreen to render it pleasant to the taste (any other oil will do). I inject this every other day or every third day, until the pockets are closed with a new growth. Where there is no necrosis of the process I use the zinc iodid solutions, one to three per cent. in distilled water. The patient must use, during the whole course of the treatment, either a hydronaphthol wash or boro-glycerol mouth-wash from five to ten times daily.

The hydronaphthol wash is made as follows:

Hydronaphthol, gr. xx;
Alcohol, oz. iii;
Oil cassia, min. iii;
Water distilled, oz. xx. M.

S.—Dilute with water if necessary.

Or a boro-glycerol wash, one ounce to twenty of distilled water. Frequent massage of the gums is recommended. Loose teeth are banded with pure silver, or silver ninety-five parts and gold five parts. Pure water is recommended, and frequent bathing is insisted upon, except in the case of the extremely aged. Personal habits of moderation are advised in all things, especially in eating and drinking. I regard a perfect occulsion as important, and accomplish this by grinding the teeth when it is demanded.

Any intercurrent constitutional malady is left to the medical adviser.

The above treatmenl, if persisted in faithfully for three months, will generally effect a cure. · If it does not, I allow the patient to go for a month, and recommend and give the case a second period of this vigorous treatment for another three months. At the end of that time the subject will appreciate the necessity for following your directions, and with his co-operation the case will yield gratifying results.—*Dental Cosmos.*

